# Effects of symbiotic bacteria on the parasitism efficacy of *Aphidius gifuensis* against *Myzus persicae*

**DOI:** 10.3389/fmicb.2026.1811839

**Published:** 2026-04-29

**Authors:** Gul Warin Khan, Gang Gu, Yufei Lai, Chen Yang, Ting Zhou, Rongquan Lai, Bang Zhang

**Affiliations:** 1 State Key Laboratory of Agriculture and Forestry Biosecurity/Biological Control Research Institute, Fujian Agriculture and Forestry University, Fuzhou, China; 2 Fujian Province Corporation of China National Tobacco Corporation, Fuzhou, China; 3 College of Plant Protection, Shandong Agricultural University, Tai’an, China

**Keywords:** *Aphidius gifuensis*, microbial symbiosis, *Myzus persicae*, parasitism rate, symbiotic bacteria

## Abstract

*Aphidius gifuensis* is an important parasitic wasp used to control *Myzus persicae* (tobacco aphid), a key pest in tobacco-producing areas. Over the past 10 years, the use of *A. gifuensis* has been widely promoted, and its natural population has steadily increased, playing a crucial role in aphid control and the prevention of aphid-transmitted viral diseases. However, long-term aphid protection and inbreeding of *A. gifuensis* populations have led to the degeneration of the species, reduced parasitism efficiency, and increased control costs. In response to these challenges, this study investigated the effects of symbiotic bacteria on the parasitism and reproductive capacity of *A. gifuensis*. The results show that *Bacillus subtilis* and *Exiguobacterium gifuensis* significantly promoted the parasitism rate of *A. gifuensis*, with the highest increases of 5.33 and 3.67%, respectively, compared to the control. Additionally, the presence of *Acinetobacter radioresistens* altered the activity of the superoxide dismutase (SOD) enzyme in *A. gifuensis*. At all tested concentrations, except for 0.97 × 10^10^ CFU/mL^–1^, SOD enzyme protein levels were inhibited, with the greatest reduction of 1.81% compared to the control. Furthermore, *A. radioresistens* significantly reduced the total activity of the SOD enzyme by 1.54%. *Bacillus subtilis* also significantly suppressed phenoloxidase activity, which was reduced by 44.78% compared to the control. These findings suggest that *B. subtilis* and *E. gifuensis* are beneficial for enhancing the parasitism efficacy of *A. gifuensis* in controlling *M. persicae*.

## 1 Introduction

Endophytes are microorganisms that colonize host tissues without causing apparent harm and are traditionally classified as plant or animal endophytes. Plant endophytes inhabit internal plant tissues without inducing visible disease symptoms, whereas animal-associated endophytes, including those found in insects, reside within specific tissues such as the gut, Malpighian tubules and reproductive organs (Aleynova and Kiselev, 2023). In insects, the composition and distribution of endophytic or symbiotic microorganisms are shaped by host species, tissue type and environmental conditions (Coolen et al., 2022). These microorganisms often establish long-term mutualistic associations that play essential roles in host nutrition, development, reproduction, and evolutionary adaptation (Holt et al., 2024). For instance, infection with *Rickettsia* enhances fecundity and offspring survival in whiteflies (Fan et al., 2022), while *Wolbachia* is known to induce *thelytokous* reproduction and increase reproductive output in several parasitoid wasps (Fricke and Lindsey, 2023).

Aphids host a diverse community of symbiotic bacteria, including the obligate primary symbiont *Buchnera aphidicola* and a range of facultative secondary symbionts such as *Rickettsia*, *Fukatsuia symbiotica*, *Rickettsiella viridis*, *Wolbachia*, *Hamiltonella defensa*, *Regiella insecticola*, *Serratia*, *Arsenophonus*, and *Spiroplasma* (Guo et al., 2017). Primary endosymbionts are vertically transmitted and provide essential amino acids and nutrients required for host growth and reproduction (Liang et al., 2024). In contrast, secondary symbionts exhibit both vertical and horizontal transmission and contribute to diverse ecological functions, including resistance to natural enemies, tolerance to environmental stress, pathogen defense and reproductive regulation (Eleftherianos et al., 2013).

The tobacco aphid, *Myzus persicae*, is a major pest in tobacco production systems and causes substantial economic losses even under natural enemy pressure (Lai et al., 2017). Aphid infestation results in honeydew secretion that promotes sooty mold development, contaminates leaves and facilitates the transmission of viral diseases such as tobacco mosaic virus (TMV) and cucumber mosaic virus (CMV), thereby reducing yield and quality (Lojek and Orlob, 1969). Although chemical, physical and biological control methods are commonly employed, the long-term reliance on chemical pesticides has led to resistance development, residue accumulation and pest resurgence, collectively referred to as the “3R problem.” Physical control strategies, including yellow sticky traps and insecticidal lamps, lack selectivity and may adversely affect beneficial insects (Ward et al., 2024).

*Aphidius gifuensis* is a dominant parasitoid of *M. persicae* and has been widely applied as a biological control agent in China’s tobacco-growing regions (Xue et al., 2023). Since 2018, its large-scale deployment has expanded to more than 14 million hectares across tobacco fields and other crops, significantly reducing pesticide use and enhancing ecological safety (Liu and Chen, 2024; Yu et al., 2021). In recognition of its success, the Food and Agriculture Organization (FAO) identified this approach as a global model for sustainable biological pest control (Xue et al., 2023). However, the low abundance of natural populations and the degeneration associated with long-term mass rearing necessitate artificial propagation and optimization strategies to maintain parasitism efficiency (Li et al., 2024).

Symbiotic microorganisms are increasingly recognized as key regulators of insect growth, development and reproductive performance (Luan, 2024). Parasitic wasps harbor a variety of microbial associates, including *Wolbachia*, *Rickettsia*, *Cardinium* and *Spiroplasma*, which can influence sex determination, fecundity, dispersal and parasitic behavior (Duron et al., 2008). *Wolbachia*, a widespread Gram-negative proteobacterium, induces reproductive manipulations such as thelytoky, sex-ratio distortion and enhanced fecundity in several hymenopteran parasitoids (Fricke and Lindsey, 2023; Zhou et al., 2023). Similarly, *Rickettsia* has been reported to induce parthenogenesis and female-biased offspring production in parasitoid species such as *Neochrysocharis formosa* and *Pnigalio soemius* (Hagimori et al., 2006). In contrast, some symbionts may negatively affect parasitoid performance by interfering with host recognition or parasitism efficiency, as observed for *Hamiltonella defensa* and *Spiroplasma* in aphid-parasitoid systems (McLean et al., 2020).

Beyond reproductive effects, symbiotic bacteria can modulate key enzymatic pathways involved in insect development, immunity and metabolism (Osborne et al., 2023). Enzymes such as carboxylesterases (COEs), phenol oxidase (PO), and superoxide dismutase (SOD) play central roles in detoxification, immune defense and oxidative stress regulation during insect growth and metamorphosis (Lu et al., 2014). COEs are involved in xenobiotic detoxification, neural development and reproductive processes (Hatfield et al., 2016), while PO participates in melanization, wound healing and immune responses (Cerenius and Söderhäll, 2021). SOD, a key antioxidant enzyme, protects cells from oxidative damage and maintains redox homeostasis (Sheng et al., 2014). Symbiont-mediated regulation of these enzymes has been reported in several insect systems, suggesting a mechanistic link between microbial associations and host physiological performance (Li et al., 2019). In this study, we investigated the effects of symbiotic bacteria on the parasitic performance, reproductive capacity and offspring sex ratio of *Aphidius gifuensis*, with a particular focus on their influence on key growth and development-related enzymes. By elucidating the interactions between symbiotic bacteria and parasitoid physiology, this work provides a scientific basis for improving the efficiency of *A. gifuensis* in the biological control of *Myzus persicae* in tobacco ecosystems.

## 2 Materials and methods

### 2.1 Test materials

Symbiotic bacteria were provided by National Key Laboratory of Biosafety for Agriculture and Forestry, Fujian Agriculture and Forestry University. The Yunyan 87 Tobacco variety was used and provided by Longyan Tobacco Company, Fujian Province. The wingless tobacco aphid was used in the experiment, provided by Shanghang Branch of Longyan Tobacco Company, which was used as the aphid source after 50 generations of indoor breeding. The aphid wasp was collected from tobacco plants in the Science and Technology Park of Shanghang Branch of Longyan Tobacco Company, and is reared under controlled conditions at Fujian Agriculture and Forestry University for 5 generations before testing.

The reagents, Nutrient broth (NA) and phosphate buffer solution (PBS, 0.04 mol/L, pH 7.0), sodium chloride (NaCl), High-purity biochemical reagents included Coomassie Brilliant Blue G-250 (100%), Fast Blue B salt, bovine serum albumin, physostigmine (98%), Sodium dodecyl sulfate (SDS) (92.5%), catechol (99.5%), riboflavin (98%), and 1-naphthyl acetate (98%), used in this study and were sourced from reliable commercial suppliers. All laboratory procedures were conducted using standard microbiological and biochemical equipment available at the Biological Control Research Institute, Fujian Agriculture and Forestry University. Analytical-grade reagents were used throughout the experiments unless otherwise specified.

### 2.2 Preparation of symbiotic bacterial cell suspension

The symbiotic bacteria were cultured in NA medium at 37°C with shaking at 230 r/min until the OD_600nm_ reached approximately 1. The bacterial cells were then harvested by centrifugation at 8,000 × g for 5 min at 4°C, washed three times with sterile 0.85% NaCl solution and resuspended in the same solution. Serial dilutions of the bacterial suspension were prepared to concentrations of 10^–1^, 10^–2^, 10^–3^, and 10^–4^ of the initial concentration. The cell density of the bacterial suspension was determined using OD_600_ measurements and CFU counts, after which infection assays were conducted (Chengpeng et al., 2025).

### 2.3 Effects of symbiotic bacteria on the parasitism rate, fecundity, and sex ratio of *Aphidius gifuensis*

Third-instar tobacco aphids, parasitized by *Aphidius gifuensis*, were transferred onto nylon mesh and immersed for 5 s in either 50 mL of a symbiotic bacterial suspension or 50 mL of sterile 0.85% NaCl solution (control). Excess liquid on the mesh was removed with filter paper, and the aphids were left to air-dry naturally before being transferred into 1.5 mL centrifuge tubes and kept in an artificial climate chamber. The symbiotic bacteria were intended to interact with the aphid’s microbiome, potentially modulating physiological traits that influence parasitoid performance. The bacteria were not intended to infect the parasitoid larvae directly but could influence parasitoid success indirectly through changes in the aphid’s physiology, such as immune function or reproductive capacity. Following emergence, female wasps were allowed to mate at a ratio of 1:50. After 12 h, *Aphidius gifuensis* adults were removed, and the aphids were maintained individually under controlled conditions. Each symbiotic bacterial strain was tested at five concentrations, along with a control, and three replicates were used for each treatment. Parasitism, emergence, sex ratio, and body size of *Aphidius gifuensis* were recorded. The parasitism rate and emergence success were calculated using Formulas 1 and 2, respectively, while hind tibia length was measured as an indicator of body size (Chengpeng et al., 2025).


$$\mathrm{Parasitism}\mathrm{rate}\left(\%\right)=\frac{\mathrm{Total}\,\mathrm{No}.\mathrm{of}\,\mathrm{parasitized}\,\mathrm{Aphids}}{\mathrm{Total}\,\mathrm{No}.\mathrm{of}\,\mathrm{aphids}\,\mathrm{exposed}}\times 100$$ (1)

$$\mathrm{Emergence}\mathrm{rate}\left(\%\right)=\frac{\mathrm{Total}\,\mathrm{No}.\mathrm{of}\,\mathrm{emerge}\,\mathrm{parasitoids}}{\mathrm{Total}\,\mathrm{No}.\mathrm{of}\,\mathrm{parasitized}\,\mathrm{Aphids}}\times 100$$ (2)

### 2.4 Effects of symbiotic bacteria on enzymes regulating growth and development in *Aphidius gifuensis*

#### 2.4.1 Treatment of test insects

Third-instar tobacco aphids parasitized by *Aphidius gifuensis* were placed on nylon mesh and immersed for 5 s in either 50 mL of a symbiotic bacterial suspension or 50 mL of sterile 0.85% NaCl solution (control). Excess liquid on the mesh was removed with filter paper and the aphids were allowed to air-dry naturally. Individual aphids were then transferred into 1.5 mL centrifuge tubes and kept in an artificial climate chamber. After emergence, female wasps were allowed to mate at a ratio of 1:50, after which the *A. gifuensis* adults were removed following 12 h. The tobacco aphids were subsequently maintained individually under controlled conditions for further observation. Each bacterial strain was tested at five concentrations plus one control, with three replicates per treatment. All experiments were initiated within 12 h of insect emergence (Chengpeng et al., 2025).

### 2.5 Determination of carboxylesterase concentration and enzymatic activity

#### 2.5.1 Preparation of standard curve

To generate a standard curve, solutions were prepared as described in Table 1, with each solution added to 6–10 mL stoppered test tubes. The tubes were capped and inverted vertically to mix the contents. The samples were left at room temperature for 5 min. Tube No. 1 was used as a blank control. Absorbance was measured at 595 nm using a colorimetric cuvette with a 1 cm light path. The OD values for each sample were recorded, and a standard curve was generated based on these measurements.

**TABLE 1:** Protein standard curve added sample table.

Test tube number	Standard protein solution (mL)	Distilled water (mL)	Coomassie brilliant blue G-250 reagent (mL)	Protein content (μ g⋅mL^–1^)
1	0	1.0	1.0	0
2	0.2	0.8	1.0	20.0
3	0.4	0.6	1.0	40.0
4	0.6	0.4	1.0	60.0
5	0.8	0.2	1.0	80.0
6	1.0	0	1.0	100.0

#### 2.5.2 Preparation of enzyme solution

For each treatment and control, 50 adults of *Aphidius gifuensis* were selected and placed into 1.5 mL centrifuge tubes, with each treatment and control replicated three times. For enzyme solution preparation, 50 adult aphids of similar size and activity were collected per treatment or control and placed in 1.5 mL centrifuge tubes. One milliliter of phosphate buffer (0.04 mol/L, pH 7.0) was added and the samples were homogenized on ice. The homogenate was then centrifuged at 10,800 r/min for 20 min at 4°C using a high-speed refrigerated centrifuge and the supernatant obtained was used as the enzyme source, with each treatment and control performed in triplicate (Zhou et al., 2021).

#### 2.5.3 Determination of carboxylesterase concentration

Using the Coomassie Brilliant Blue G-250 method, 6–10 mL stoppered test tubes were prepared. One milliliter of the enzyme sample was added to each tube, followed by 1 mL of Coomassie Brilliant Blue G-250 reagent. The tubes were gently inverted several times to mix thoroughly and left at room temperature for 5 min. Absorbance was then measured at 595 nm using a colorimetric cuvette with a 1 cm light path. The OD values for each tube were recorded and carboxylesterase protein content was calculated based on a standard curve (Bradford, 1976).

#### 2.5.4 Carboxylesterase activity assay

A reaction mixture of 3.2 mL was prepared, including 450 μL of 0.04 mol/L phosphate buffer (pH 7.0), 1.8 mL of substrate composed of 1-naphthyl acetate (3 × 10^–4^ mol/L) and physostigmine (1 × 10^–4^ mol/L) mixed in equal parts and 50 μL of the enzyme solution. The mixture was incubated in a water bath at 30°C for 15 min. To stop the reaction, 0.9 mL of a color-developing solution made by combining (1% Fast Blue B salt and 5% SDS in a 2:5 ratio) was added. After standing for 15 min, the absorbance at 600 nm was recorded. The specific activity of the enzyme was calculated using these absorbance values and the protein concentration of the enzyme extract (van Asperen, 1962; Yang, 2016). Using the recorded OD values and the enzyme solution’s protein content, the specific enzyme activity was calculated according to Formula 3.


$$\mathrm{Specific}\,\mathrm{Activity}=\frac{O\,D\times V}{t\times \rho_{\text{pro}}\times V\,\text{e}}$$ (3)

In the formula:

OD—Absorbance was measured at the specified wavelength and expressed in terms of naphthol content (μmoL).

V—Total volume of the reaction mixture (mL).

t—Duration of the reaction (minutes).

ρ_pro_—Concentration of protein in enzyme solution (mg/mL).

V_e_—Enzyme solutions volume used in the reaction (mL).

### 2.6 Determination of phenoloxidase concentration and function

#### 2.6.1 Preparation of enzyme solution

For each treatment and control, 50 adults of *Aphidius gifuensis* of comparable size and activity were chosen and placed in 1.5 mL centrifuge tubes. To each tube, 1 mL of phosphate buffer (0.2 mol/L, pH 6.9) was added, and the samples were homogenized on ice. The homogenates were allowed to rest for 15 min in an ice bath, then centrifuged at 8,000 rpm for 30 min at 4°C in a high-speed refrigerated centrifuge. Surface lipids and pigments were removed, and the resulting supernatant was collected as the enzyme source for subsequent assays. Each treatment and control was replicated three times (Jing et al., 2022; Zhu et al., 2015).

#### 2.6.2 Determination of phenoloxidase concentration

Using the Coomassie Brilliant Blue G-250 method, 6–10 mL stoppered test tubes were prepared. A total of 1 mL of the enzyme sample was added to each tube, followed by 1 mL of Coomassie Brilliant Blue G-250 reagent. The tubes were gently inverted several times to mix thoroughly and left at room temperature for 5 min. Absorbance was then measured at 595 nm using a colorimetric cuvette with a 1 cm light path. The OD values for each tube were recorded, and carboxylesterase protein content was calculated based on a standard curve (Bradford, 1976).

#### 2.6.3 Phenol oxidase activity assay

A reaction mixture with a total volume of 2 mL was prepared by combining 1 mL of phosphate buffer (0.1 mol/L, pH 7.0) with 700 μL of 1 U mol/L catechol solution. Then, 300 μL of the enzyme solution was added and gently mixed for 5 s, using the buffer alone as a reference. Absorbance at 475 nm (OD_475_) was measured continuously for 3 min to track changes over time and construct an optical density growth curve. One unit of enzyme activity was defined as an increase of 0.001 in OD_475_ per minute (Jian et al., 2025). Enzyme activity was calculated using Formula 4.


$$\mathrm{Enzyme}\,\mathrm{activity}=\frac{\Delta \,A\,475\times V\,t}{W\times V\,S\times 0.001\times t}$$ (4)

In the formula:

V_e_—Total enzyme volume extract (mL).

W—Mass of *Aphidius gifuensis* adults (mg).

V_e_—Volume of enzyme solution used in the reaction (mL).

t—Duration of the reaction (minutes).

### 2.7 Determination of superoxide dismutase concentration and activity

#### 2.7.1 Preparation of enzyme solution

For each experimental group and the control, 50 adults of aphids with comparable size and activity were placed into a 1.5 mL centrifuge tube. One milliliter of ice-cold PBS buffer (0.1 mol/L, pH 7.0) was then added and the insects were homogenized while kept at a low temperature. The homogenate was then centrifuged at 16,500 × g for 20 min at 4°C using a high-speed refrigerated centrifuge. The collected supernatant was designated as the enzyme solution for assessing SOD activity, with triplicate replications applied to both treatments and controls (Hui et al., 2006).

#### 2.7.2 Determination of superoxide dismutase concentration

Using the Coomassie Brilliant Blue G-250 method, 6–10 mL stoppered test tubes were prepared. One milliliter of the enzyme sample was added to each tube, followed by 1 mL of Coomassie Brilliant Blue G-250 reagent. The tubes were gently inverted several times to mix thoroughly and left at room temperature for 5 min. Absorbance was then measured at 595 nm using a colorimetric cuvette with a 1 cm light path. The OD values for each tube were recorded and carboxylesterase protein content was calculated based on a standard curve (Bradford, 1976).

#### 2.7.3 Superoxide dismutase activity assay

A 3 mL reaction system was prepared, consisting of 1.5 mL phosphate-buffered saline (0.1 mol/L, pH 7.0), 300 μL methionine solution (13 mmol/L), 300 μL riboflavin (80 μmol/L), 300 μL nitroblue tetrazolium (77 μmol/L), 300 μL EDTA (0.1 mmol/L), and 100 μL of the enzyme extract. Two control tubes were included: one containing only buffer and kept in the dark and the other treated under the same conditions as the samples. After mixing, the sample tubes were evenly illuminated at 4,000 lx for 5 min, while the dark control tube served as a blank. The reaction was then stopped by shielding the tubes from light. The absorbance of the illuminated reaction mixtures was recorded at 560 nm (OD_560_) using the NBT assay method (Beauchamp and Fridovich, 1971). All treatments and controls were performed in triplicate. One unit (U) of SOD activity was defined as the enzyme quantity required to inhibit nitroblue tetrazolium reduction by 50%. The total SOD activity was then determined according to Formula 5.


$$\mathrm{Total}\,\mathrm{SOD}\,\mathrm{activity}=\frac{\left(A_{\text{CK}}-A_{E}\right)\times V}{0.5\times A_{C\,K}\times W\times V_{t}}$$ (5)

In the formula:

A_*kk*_—Absorbance of the illuminated control tube.

A_e_—Absorbance measured for sample tube.

V—Total volume of the sample (mL).

W—Mass of the tobacco aphids used (mg).

V_t_—Sample volume used in the reaction (mL).

### 2.8 Data analysis

Data on parasitism rate, emergence rate, sex ratio, lifespan, body size and growth-related enzyme activities of *Aphidius gifuensis* were recorded, organized, microsoft Excel 2016 was used for data processing, while Two-way ANOVA was carried out in SPSS 22.0 and Duncan’s multiple range test was applied for *post-hoc* comparisons. All results are presented as mean ± standard error (SE). Graphs were generated using GraphPad Prism 8.0.2.

## 3 Results

### 3.1 Effects of symbiotic bacteria on parasitism, emergence, sex ratio, and morphometric traits of *Aphidius gifuensis*

#### 3.1.1 Effects of *Bacillus subtilis* and *Lysinibacillus fusiformis* on parasitism rate, emergence rate, sex ratio, and morphometric traits

**FIGURE 1:**
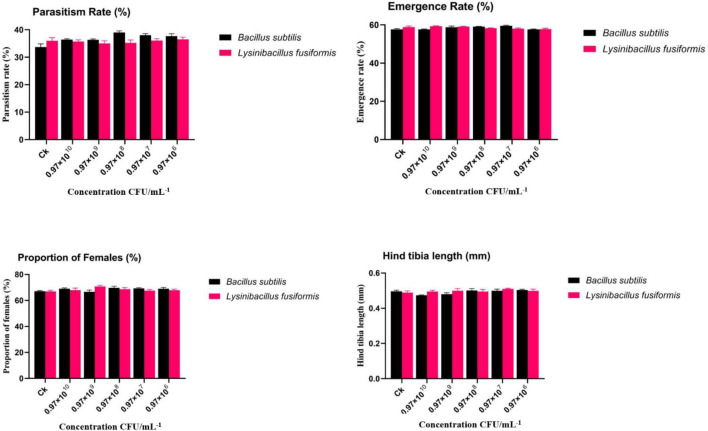
Effects of *Bacillus subtilis* and *Lysinibacillus fusiformis* on the biological performance of *Aphidius gifuensis*. Parasitism rate, emergence rate, proportion of females, and hind tibia length at different bacterial concentrations (10^6^–10^10^ CFU mL^–1^). Values represent mean ± SE (*n* = 3). Different lowercase letters indicate significant differences among treatments according to Duncan’s multiple range test (*p* < 0.05).

As shown in Figure 1, the biological performance of *Aphidius gifuensis* was evaluated across concentrations ranging from 10^6^ to 10^10^ CFU/mL^–1^. The parasitism rate (%) varied from 33.6 ± 1.2% in the control (CK) to 36.6 ± 0.8% across all treatments. For *Bacillus subtilis*, parasitism ranged from 36.43 ± 0.3% at 0.97 × 10^10^ CFU/mL^–1^ to 38.9 ± 0.5% at 0.97 × 108 CFU/mL^–1^. For *Lysinibacillus fusiformis*, parasitism rates ranged from 35.7 ± 0.5% at 0.97 × 10^10^ CFU/mL^–1^ to 35.2 ± 0.9% at 0.97 × 108 CFU/mL^–1^. The emergence rate was consistently high, ranging from 57.6 ± 0.3% in the control (CK) to 57.8 ± 0.5% across treatments. In *Bacillus subtilis* treatments, emergence ranged from 57.6 ± 0.1% to 59.2 ± 0.2%, and in *Lysinibacillus fusiformis* treatments, values ranged from 59.4 ± 0.2% to 58.8 ± 0.2%. The proportion of females remained stable across treatments, varying from 67.2 ± 0.5% in the control (CK) to 67.9 ± 0.8%. For *Bacillus subtilis*, values ranged between 68.8 ± 0.6% and 69.6 ± 1.3%, and for *Lysinibacillus fusiformis*, values ranged from 67.9 ± 1.0% to 68.7 ± 1.2%. The hind tibia length (mm), a measure of morphometric traits, showed minimal variation across treatments, ranging from 0.46 ± 0.01 mm in the control (CK) to 0.50 ± 0.01 mm. For *Bacillus subtilis*, tibia length ranged from 0.49 ± 0.01 mm to 0.49 ± 0.01 mm, while for *Lysinibacillus fusiformis*, tibia length ranged from 0.47 ± 0.01 mm to 0.50 ± 0.01 mm.

#### 3.1.2 Effects of *Bacillus pumilus* and *Acinetobacter radioresistens* on parasitism rate, emergence rate, sex ratio, and morphometric traits

**FIGURE 2:**
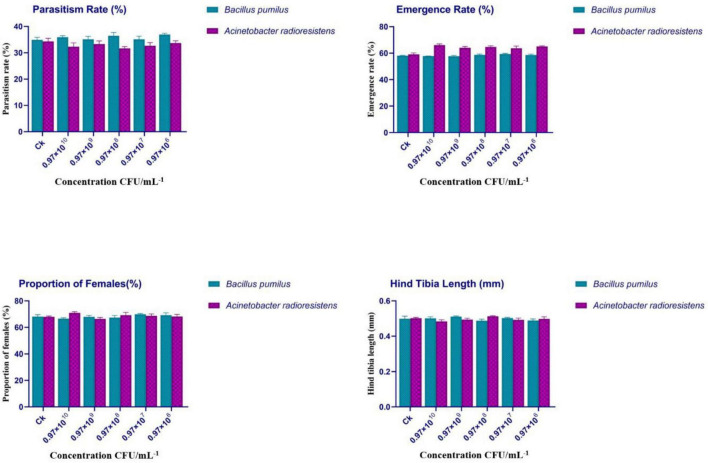
Effects of *Bacillus pumilus* and *Acinetobacter radioresistens* on the biological performance of *Aphidius gifuensis*. Parasitism rate, emergence rate, proportion of females, and hind tibia length across different bacterial concentrations (10^6^–10^10^ CFU mL^–1^). Values represent mean ± SE (*n* = 3). Different lowercase letters indicate significant differences among treatments according to Duncan’s multiple range test (*p* < 0.05).

As shown in Figure 2, the biological performance of *Aphidius gifuensis* was evaluated across concentrations ranging from 10^6^ to 10^10^ CFU/mL^–1^. The parasitism rate (%) exhibited values between 34.6 ± 0.9% in the control (CK) to 33.6 ± 0.8% across all treatments. For *B. pumilus*, parasitism ranged from 35.9 ± 0.9% at 0.97 × 10^10^ CFU/mL^–1^ to 36.4 ± 1.2% at 0.97 × 108 CFU/mL^–1^. For *A. radioresistens*, parasitism ranged from 32.3 ± 1.4% at 0.97 × 10^10^ CFU/mL^–1^ to 31.6 ± 0.6% at 0.97 × 10^8^ CFU/mL^–1^. The emergence rate (%) was consistently high, ranging from 58.1 ± 0.3% in the control (CK) to 65.1 ± 0.5% across treatments. In *B. pumilus* treatments, emergence varied between 57.6 ± 0.1% and 58.6 ± 0.6%, whereas in *A. radioresistens* treatments, values ranged from 66.1 ± 1.0% to 64.8 ± 0.8%. The proportion of females (%) was stable across treatments, varying from 68.1 ± 1.4% in the control (CK) to 68.2 ± 1.5% across treatments. For *B. pumilus*, values ranged between 66.5 ± 0.6% and 67.3 ± 1.5%, and for *A. radioresistens*, values ranged from 70.9 ± 0.9% to 69.2 ± 2.0%. The hind tibia length (mm) showed minimal variation among treatments, ranging from 0.49 ± 0.01 mm in the control (CK) to 0.48 ± 0.01 mm. For *B. pumilus*, values ranged between 0.51 ± 0.01 mm and 0.48 ± 0.01 mm, while for *A. radioresistens*, values ranged from 0.47 ± 0.01 mm to 0.51 ± 0.01 mm.

#### 3.1.3 Effects of *Exiguobacterium Sibiricum* and *Bacillus cereus* on parasitism rate, emergence rate, sex ratio, and morphometric traits

**FIGURE 3:**
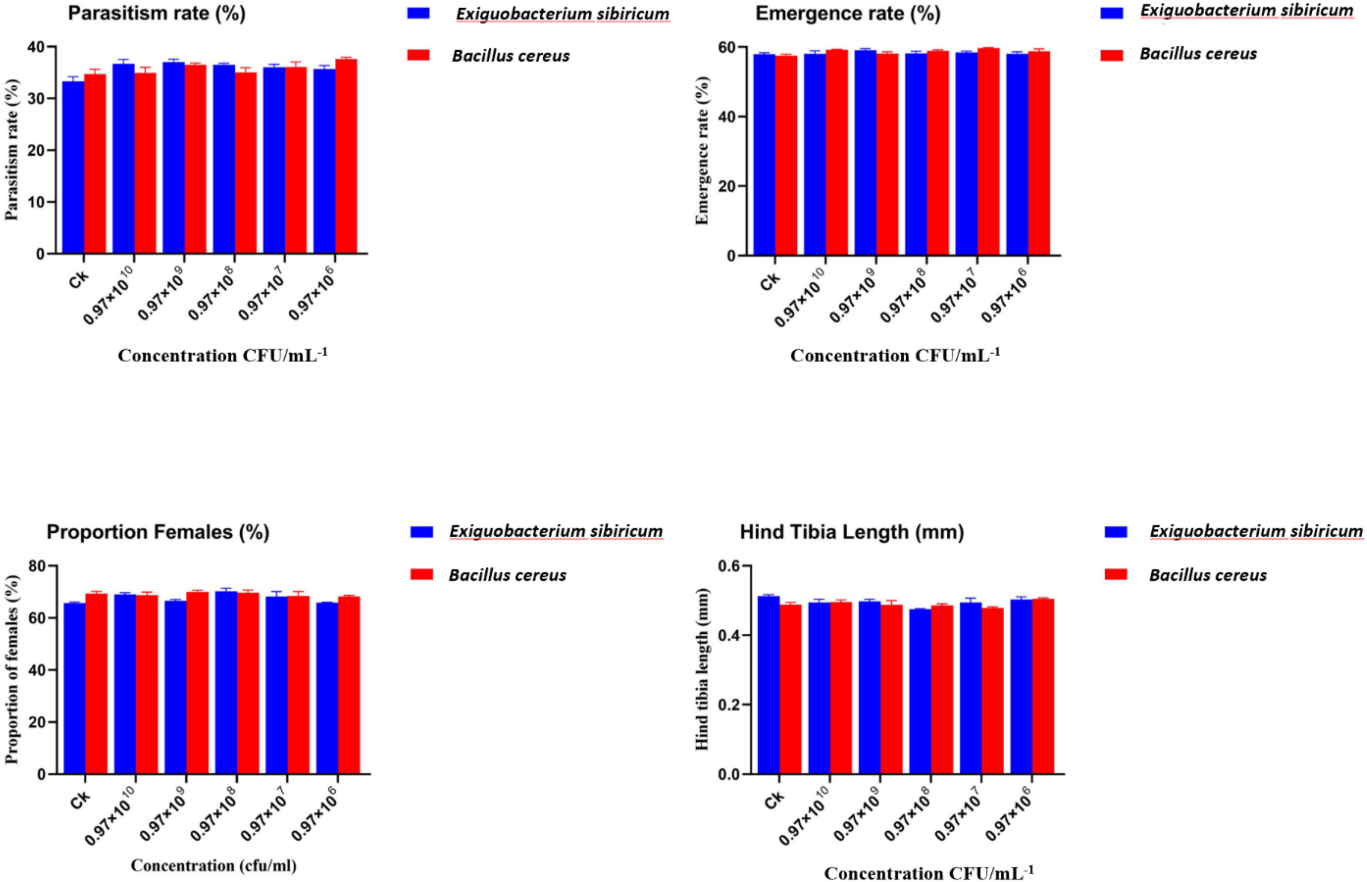
Effects of *Exiguobacterium sibiricum* and *Bacillus cereus* on the biological performance of *Aphidius gifuensis*. Parasitism rate, emergence rate, proportion of females, and hind tibia length under different bacterial concentrations (10^6^–10^10^ CFU mL^–1^). Values represent mean ± SE (*n* = 3). Different lowercase letters indicate significant differences among treatments according to Duncan’s multiple range test (*p* < 0.05).

As shown in Figure 3, the biological performance of *Aphidius gifuensis* was evaluated across concentrations ranging from 10^6^ to 10^10^ CFU/mL^–1^. The parasitism rate (%) exhibited values between 33.3 ± 0.8% in the control (CK) to 37.6 ± 0.3% across all treatments. For *E. Sibiricum*, parasitism ranged from 36.6 ± 0.8% at 0.97 × 10^10^ CFU/mL^–1^ to 36.5 ± 0.2% at 0.97 × 10^8^ CFU/mL^–1^. For *B. cereus*, parasitism ranged from 34.9 ± 1.1% at 0.97 × 10^10^ CFU/mL^–1^ to 35.1 ± 0.9% at 0.97 × 10^8^ CFU/mL^–1^. The emergence rate (%) was consistently high, ranging from 57.9 ± 0.4% in the control (CK) to 65.1 ± 0.5%. In *E. Sibiricum* treatments, emergence varied between 58.1 ± 0.8% and 58.1 ± 0.6%, whereas in *B. cereus* treatments, values ranged from 59.1 ± 0.1% to 58.8 ± 0.3%. The proportion of females (%) was stable across treatments, varying from 65.6 ± 0.4% in the control (CK) to 68.2 ± 0.5%. For *E. Sibiricum*, values ranged between 68.9 ± 0.7% and 70.1 ± 1.2%, and for *B. cereus*, values ranged from 68.7 ± 1.1% to 69.7 ± 0.9%. The hind tibia length (mm) showed minimal variation among treatments, ranging from 0.50 ± 0.01 mm in the control (CK) to 0.51 ± 0.01 mm. For *E. Sibiricum*, values ranged between 0.49 ± 0.01 mm and 0.47 ± 0.01 mm, while for *B. cereus*, values ranged from 0.49 ± 0.01 mm to 0.48 ± 0.01 mm.

#### 3.1.4 Effects of *Stenotrophomonas maltophilia* on parasitism rate, emergence rate, sex ratio, and morphometric traits

As shown in Table 2 show, effects of different concentrations of the tested treatment (10^6^ – 10^10^ CFU/mL^–1^) on the biological parameters of the parasitoid were analyzed and compared with the control (Ck). The results showed that parasitism rate remained relatively stable across treatments, ranging from 35.9 ± 0.5% to 35.1 ± 0.8%, with no significant difference compared to the control (35.40 ± 1.2%). Similarly, emergence rate varied only slightly (58.23 ± 0.6 to 58.00 ± 0.3%) and did not differ significantly among treatments. The proportion of females also showed minor variation (68.13 ± 1.07 to 67.9 ± 1.4%), with values comparable to the control. In terms of morphological traits, hind tibia length ranged from 0.48 ± 0.01 to 0.498 ± 0.01 mm across treatments, again with no significant differences relative to the control (0.497 ± 0.01 mm). Overall, these findings indicate that exposure to different concentrations of the tested agent had no significant impact on parasitism efficiency, emergence success, sex ratio, or tibia length of the parasitoid.

**TABLE 2:** Effect of *S. maltophilia* on the parasitism rates emergence rate, proportion of females and body size of *A. gifuensis* adults.

Concentrantion (CFU/mL^–1^)	Parasitism rate (%)	Emergence rate (%)	Proportion of females (%)	Hind tibia length (mm)
Ck	35.40 ± 1.22a	58.60 ± 0.71a	69.27 ± 0.89a	0.497 ± 0.008a
0.97 × 10^10^	35.93 ± 0.50a	58.23 ± 0.62a	68.13 ± 1.07a	0.487 ± 0.006a
0.97 × 10^9^	36.10 ± 1.55a	58.73 ± 0.83a	68.80 ± 0.55a	0.480 ± 0.007a
0.97 × 10^8^	35.07 ± 0.83a	58.00 ± 0.31a	67.97 ± 1.48a	0.498 ± 0.011a
0.97 × 10^7^	36.13 ± 1.14a	58.53 ± 0.62a	67.27 ± 1.16a	0.498 ± 0.005a
0.97 × 10^6^	35.40 ± 1.36a	58.33 ± 0.60a	68.40 ± 0.12a	0.493 ± 0.010a

The letters represent statistical groupings based on the results of an ANOVA test. Different letters indicate significant differences between the groups.

### 3.2 Impact of symbiotic bacteria on enzymes regulating the growth and development of *Aphidius gifuensis*

Three symbiotic bacteria (*A. radioresistens*, *B. subtilis*, and *E. truncatum*) which respectively influenced the parasitism rate, emergence rate and female offspring ratio of *A. gifuensis*, were assessed for their role in modulating enzymes involved in the growth and development of *A. gifuensis* (Tables 3–5).

**TABLE 3:** Effects of *Acinetobacter radioresistens* on growth-related enzyme content and activity (carboxylesterase, phenoloxidase, and SOD) in *Aphidius gifuensis* offspring.

Concentrantion (CFU/mL^–1^)	Carboxylesterase content (mg/mL)	Carboxylesterase activity μ moL/(min⋅mg)	Phenoloxidase content (mg/mL)	Phenoloxidase activity U/(mg⋅min)	SOD content (mg/mL)	SOD activity U/(mg⋅min)
Ck	0.0894 ± 0.0002a	2.5133 ± 0.0450a	0.0933 ± 0.0003a	4.0564 ± 0.7055ab	0.0827 ± 0.0002c	12.4216 ± 0.0055c
0.97 × 10^10^	0.0887 ± 0.0004a	2.5817 ± 0.1022a	0.0935 ± 0.0009a	3.1746 ± 0.6110ab	0.0828 ± 0.0002c	12.3902 ± 0.0175c
0.97 × 10^9^	0.0886 ± 0.0005a	2.6487 ± 0.1561a	0.0931 ± 0.0007a	4.5855 ± 0.1764b	0.0815 ± 0.0007ab	12.2306 ± 0.0261a
0.97 × 10^8^	0.0891 ± 0.0003a	2.5841 ± 0.0919a	0.0922 ± 0.0007a	2.2928 ± 0.1764a	0.0816 ± 0.0004abc	12.3679 ± 0.0380c
0.97 × 10^7^	0.0889 ± 0.0005a	2.4633 ± 0.0302a	0.0923 ± 0.0003a	4.2328 ± 0.8082ab	0.0826 ± 0.0002bc	12.2816 ± 0.0285ab
0.97 × 10^6^	0.0892 ± 0.0004a	2.6141 ± 0.0244a	0.0928 ± 0.0003a	4.0564 ± 0.7688ab	0.0809 ± 0.0003a	12.3417 ± 0.0156bc

The letters represent statistical groupings based on the results of an ANOVA test. Different letters indicate significant differences between the groups.

**TABLE 4:** Effects of *Bacillus subtilis* on growth-related enzyme content and activity (carboxylesterase, phenoloxidase, and SOD) in *Aphidius gifuensis* offspring.

Concentrantion (CFU/mL^–1^)	Carboxylesterase content (mg/mL)	Carboxylesterase activity μ moL/(min⋅mg)	Phenoloxidase content (mg/mL)	Phenoloxidase activity U/(mg⋅min)	SOD content (mg/mL)	SOD activity U/(mg⋅min)
Ck	0.0898 ± 0.0001a	2.6932 ± 0.0147a	0.0949 ± 0.0001b	4.0564 ± 0.2373c	0.0823 ± 0.0007a	12.3794 ± 0.0385a
0.97 × 10^10^	0.0897 ± 0.0004a	2.6631 ± 0.0372a	0.0941 ± 0.0002ab	4.0035 ± 0.1682c	0.0824 ± 0.0002a	12.3891 ± 0.0601a
0.97 × 10^9^	0.0899 ± 0.0004a	2.6903 ± 0.0232a	0.0943 ± 0.0005ab	2.2399 ± 0.3120a	0.0831 ± 0.0007a	12.3489 ± 0.0684a
0.97 × 10^8^	0.0902 ± 0.0003a	2.6653 ± 0.0160a	0.0944 ± 0.0004ab	2.7866 ± 0.1411ab	0.0831 ± 0.0003a	12.3435 ± 0.0273a
0.97 × 10^7^	0.0896 ± 0.0005a	2.6830 ± 0.0466a	0.0943 ± 0.0003ab	2.9101 ± 0.3218ab	0.0820 ± 0.0006a	12.3673 ± 0.0445a
0.97 × 10^6^	0.0898 ± 0.0004a	2.6600 ± 0.0158a	0.0938 ± 0.0003a	3.4568 ± 0.5267bc	0.0827 ± 0.0004a	12.3707 ± 0.0096a

The letters represent statistical groupings based on the results of an ANOVA test. Different letters indicate significant differences between the groups.

**TABLE 5:** Effects of *Exiguobacterium truncatum* on growth-related enzyme content and activity (carboxylesterase, phenoloxidase, and SOD) in *Aphidius gifuensis* offspring.

Concentrantion (CFU/mL^–1^)	Carboxylesterase content (mg/mL)	Carboxylesterase activity μ moL/(min⋅mg)	Phenoloxidase content (mg/mL)	Phenoloxidase activity U/(mg⋅min)	SOD content (mg/mL)	SOD activity U/(mg⋅min)
Ck	0.6980 ± 0.0015abc	2.7229 ± 0.0204b	0.0949 ± 0.0002a	3.7037 ± 0.3055a	0.0817 ± 0.0004a	12.3659 ± 0.0490a
0.97 × 10^10^	0.7010 ± 0.0025c	2.6479 ± 0.0126a	0.0951 ± 0.0002a	3.7037 ± 0.6110a	0.0818 ± 0.0001a	12.3699 ± 0.0323a
0.97 × 10^9^	0.6947 ± 0.0019ab	2.7207 ± 0.0148b	0.0949 ± 0.0000a	4.4092 ± 0.9332a	0.0818 ± 0.0004a	12.4201 ± 0.0508a
0.97 × 10^8^	0.6930 ± 0.0017a	2.6960 ± 0.0222ab	0.0947 ± 0.0001a	4.4092 ± 1.1565a	0.0818 ± 0.0004a	12.4672 ± 0.0124a
0.97 × 10^7^	0.7000 ± 0.0010bc	2.6990 ± 0.0141ab	0.0949 ± 0.0003a	4.5855 ± 0.7688a	0.0813 ± 0.0002a	12.4185 ± 0.0407a
0.97 × 10^6^	0.6997 ± 0.0015bc	2.6690 ± 0.0174ab	0.0948 ± 0.0003a	5.1146 ± 0.4666a	0.0815 ± 0.0003a	12.3670 ± 0.0572a

The letters represent statistical groupings based on the results of an ANOVA test. Different letters indicate significant differences between the groups.

As shown in Table 3, *A. radioresistens* did not significantly influence the carboxylesterase content (*F*_5,__12_ = 0.681, *p*< 0.05) and enzyme specific activity (*F*_5,__12_ = 0.591, *p*< 0.05) of *A. gifuensis*, the phenoloxidase content (*F*_5,__12_ = 0.806, *p*< 0.05) and enzyme activity (*F*_5,__12_ = 1.969, *p*< 0.05). *A. radioresistens* significantly influenced the SOD enzyme content in *A. gifuensis* (*F*_5,__12_ = 4.765, *p*< 0.05). Except at 0.97 × 10^10^ CFU/mL^–1^ concentration of, the bacterium generally reduced SOD protein levels. At 0.97 × 10^10^ CFU/mL^–1^ concentration, the highest SOD content was observed at 0.0828 mg/mL slightly above the control by 0.0001 mg/mL, representing a 0.12% increase. In contrast, at 0.97 × 10^6^ CFU/mL^–1^, SOD content was lowest at 0.0809 mg/mL, 0.0015 mg/mL below the control, a decrease of 1.81%. Similarly, *A. radioresistens* significantly inhibited total SOD enzyme activity (*F*_5,__12_ = 8.632, *p*< 0.01). The highest activity, observed at 0.97 × 10^10^ CFU/mL^–1^, was 12.3902 U/(mg⋅min), showing a decrease of 0.0314 U/(mg⋅min) compared to the control, a 0.25% decrease. The lowest activity occurred at 0.97 × 10^7^ CFU/mL^–1^, measuring 12.2306 U/(mg⋅min), 0.1910 U/(mg⋅min) less than the control, representing a 1.54% reduction.

As shown in Table 4, the carboxylesterase content was not significantly affected by *Bacillus subtilis* (*F*_5,__12_ = 0.299, *p*< 0.05) or specific enzyme activity (*F*_5,__12_ = 0.270, *p*< 0.05) in *A. gifuensis*. It also did not significantly affect phenoloxidase content (*F*_5,__12_ = 1.420, *p*< 0.05), but it significantly inhibited phenoloxidase activity (*F*_5,__12_ = 5.376, *p*< 0.01). At 0.97 × 10^10^ CFU/mL^–1^ concentration, phenoloxidase activity was highest at 4.0035 U/(mg⋅min), showed a slight reduction compared with the control by 0.0529 U/(mg⋅min), representing a 1.30% increase. Conversely, at 0.97 × 10^10^ CFU/mL^–1^ concentration, activity was lowest at 2.2399 U/(mg⋅min), 1.8165 U/(mg⋅min) below the control, a 44.78% decrease. *B. subtilis* did not significantly affect SOD enzyme content (*F*_5,__12_ = 0.727, *p*< 0.05) or total SOD activity (*F*_5,__12_ = 0.146, *p*< 0.05) in *A. gifuensis*.

As shown in Table 5, *Exiguobacterium* significantly affected carboxylesterase content in *A. gifuensis* (*F*_5,__12_ = 3.387, *p*< 0.05). At 0.97 × 10^10^ CFU/mL^–1^ concentration, the maximum carboxylesterase content was observed at 0.7010 mg/mL, 0.0030 mg/mL above the control, representing a 0.42% increase. At 0.97 × 10^8^ CFU/mL^–1^ concentration, the lowest content observed was 0.6930 mg/mL, 0.0050 mg/mL below the control, a 0.72% decrease. However, *Exiguobacterium* had no significant effect on carboxylesterase specific activity (*F*_5,__12_ = 2.897, *p*< 0.05). *Exiguobacterium* had no significant effect on phenoloxidase content (*F*_5,__12_ = 0.402, *p*< 0.05) or activity (F_5,12_ = 0.509, *p*< 0.05), nor on SOD enzyme content (*F*_5,__12_ = 0.372, *p*< .05) or total SOD activity (*F*_5,__12_ = 0.909, *p*< 0.05) in *A. gifuensis*.

## 4 Discussion

The present study demonstrates that symbiotic bacteria exert measurable effects on the reproductive performance, parasitism efficiency, and enzymatic physiology of *Aphidius gifuensis*, an important biological control agent against *Myzus persicae*. Among the tested strains, *Bacillus subtilis* significantly enhanced both the parasitism rate and emergence success, while *Exiguobacterium gifuensis* improved parasitism and increased the proportion of female offspring. Conversely, *Acinetobacter radioresistens* exerted a dual influence: although it promoted emergence at certain concentrations, it also reduced superoxide dismutase (SOD) activity, suggesting a physiological cost. Enzymatic assays further revealed that *B. subtilis* inhibited phenoloxidase (PO) activity, and *Exiguobacterium* increased carboxylesterase (COE) content, highlighting that symbiotic bacteria not only affect host reproductive traits but also interfere with key enzyme systems involved in immunity, detoxification, and oxidative balance. These results underscore the complex interactions between microbial associates and parasitoid performance, which could have significant implications for improving biological control efficacy in tobacco systems.

While the reported increases in parasitism (5.33%) and enzymatic changes (1.81% decrease in SOD and 1.54% decline in SOD activity) are modest, these findings are biologically relevant within the context of integrated pest management (IPM). In real-world pest control applications, even small improvements in parasitoid efficiency can lead to long-term cumulative effects, enhancing pest suppression. Despite their modest magnitude, these changes indicate that microbial manipulation could be a viable method for enhancing the efficacy of *Aphidius gifuensis* in biological control programs.

The influence of symbiotic bacteria on insect physiology and reproduction is well-documented, and our findings align with broader patterns observed in other insect-microbe interactions. In aphids, secondary symbionts such as *Rickettsia and Hamiltonella defensa* have been shown to modulate host fecundity, stress tolerance, and resistance to parasitoids (Tougeron and Iltis, 2022). For instance, *Rickettsia* infection increases female-biased reproduction in *Bemisia tabaci*, enhancing population growth under favorable conditions (Liu et al., 2024). Similarly, Wolbachia is well-known for inducing *thelytokous* reproduction and skewing sex ratios toward females in parasitoid wasps such as *Trichogramma* spp. *and Nasonia vitripennis* (Fricke and Lindsey, 2023). Our observation that *Exiguobacterium* infection increased the proportion of female offspring in *Aphidius gifuensis* supports the hypothesis that symbiont-driven reproductive manipulation is a common strategy across taxa.

Our findings also resonate with studies highlighting enzyme-level regulation by symbiotic bacteria. For instance, *Hamiltonella defensa* has been shown to alter polyphenol oxidase activity in aphids, influencing immune defense mechanisms (Li et al., 2019). In our study, *B. subtilis* significantly reduced PO activity in *Aphidius gifuensis*, which may weaken immune responsiveness but simultaneously channel more resources toward reproduction. Similarly, *Acinetobacter radioresistens* suppressed SOD activity, consistent with earlier reports where microbial infections impaired antioxidant defense in insects, leading to altered stress physiology (González-Tokman et al., 2025). These findings suggest that modulation of host enzymatic pathways by microbial associates is a recurrent mechanism influencing insect performance, rather than a coincidental effect.

Despite these parallels, some differences emerge. While *Wolbachia* often enhances parasitoid dispersal and fecundity across generations, *A. radioresistens* exhibited inhibitory effects at multiple concentrations, reducing enzymatic activity and parasitism rates (Fytrou et al., 2006). This discrepancy may stem from bacterial strain-specific effects, host–microbe compatibility, or trade-offs between reproduction and immunity. Such complexity underscores the importance of evaluating a broad spectrum of microbial associates rather than assuming uniform benefits. The mechanisms by which symbiotic bacteria influence parasitoid performance are likely multifaceted, involving both direct physiological modulation and indirect metabolic trade-offs. One possible explanation for the enhanced parasitism and emergence rates observed with *B. subtilis* is its capacity to modulate nutrient assimilation or hormonal signaling in the parasitoid, improving developmental success (Vasantha-Srinivasan et al., 2025). The concurrent inhibition of PO activity suggests a reallocation of energetic resources away from immune defense toward reproduction, reflecting a classical trade-off between survival and fecundity (Martin and Tate, 2024). Reduced immunity may render parasitoids more vulnerable to pathogens; however, in controlled rearing environments, this compromise could translate into higher reproductive output.

In contrast, the effects of *A. radioresistens* on SOD activity illustrate how oxidative stress pathways may shape parasitoid fitness. SOD is critical for neutralizing reactive oxygen species (ROS) and maintaining cellular homeostasis. Suppression of SOD activity may impair oxidative balance, leading to subtle reductions in longevity or fecundity, even if short-term emergence is improved (Wang et al., 2018). The dose-dependent pattern observed in our study, where high bacterial concentrations slightly increased SOD content but lower concentrations caused inhibition, suggests a threshold-dependent response (Mittra et al., 2017). This phenomenon aligns with hormesis theory, where low levels of stressors can stimulate defense responses, while higher exposures lead to detrimental effects.

The increase in carboxylesterase content under *Exiguobacterium* treatment indicates another layer of physiological modulation. COEs are crucial in detoxification and reproduction, and their upregulation could enhance parasitoid adaptability in environments rich in plant secondary metabolites or pesticide residues (Chamani et al., 2025). Importantly, the observed increase in female offspring proportion under *Exiguobacterium* aligns with the idea that elevated COE activity may favor resource allocation toward egg development. These results collectively emphasize that symbiont-parasitoid interactions involve coordinated changes in enzymatic systems, ultimately translating into reproductive advantages or trade-offs.

From an applied perspective, the findings highlight the potential of manipulating microbial associates to improve the performance of parasitoid wasps in IPM. *A. gifuensis* has been deployed extensively in tobacco-producing regions of China, covering millions of hectares, yet its mass rearing and field efficacy are constrained by reduced parasitism under long-term breeding (Xue et al., 2023). Our study suggests that supplementation with specific microbial associates such as *B. subtilis* or *Exiguobacterium* could enhance parasitism rates and increase female-biased offspring production, thereby boosting the efficiency of mass-released populations. These microbial partners could act as natural enhancers, reducing the need for chemical inputs and aligning with sustainable agriculture goals.

Moreover, enzyme-level modulation offers a potential biomarker for monitoring parasitoid quality in rearing programs. For example, reduced PO activity may indicate compromised immunity, whereas elevated COE levels could predict higher fecundity (Leyria et al., 2025). Such physiological markers could be integrated into quality-control protocols for commercial production of parasitoids (Pobożniak and Olczyk, 2025). Additionally, the ability of symbiotic bacteria to alter oxidative stress responses highlights opportunities for selecting bacterial strains that enhance stress tolerance during transportation and release, improving survival in diverse field conditions.

Despite the promising insights, several limitations warrant consideration. First, the experiments were conducted under controlled laboratory conditions, which may not fully capture the ecological variability and microbial diversity present in the field (Chen et al., 2023). Symbiotic bacteria interact not only with parasitoids but also with host plants and aphids. These tripartite interactions may modulate outcomes in more complex ways than observed here (Azevedo et al., 2016). Second, the study tested a limited set of bacterial strains. Natural populations of parasitoids likely harbor a greater diversity of microbial associates, and the ecological roles of many remain unexplored (Tyc et al., 2020). Third, the mechanistic basis of the observed effects was restricted to enzyme assays. While these provide valuable physiological insights, molecular analyses such as transcriptomics, proteomics, and metabolomics are needed to unravel the signaling pathways and gene networks involved (Liu et al., 2025). Finally, potential long-term trade-offs, such as reduced immunity or shortened lifespan, were not assessed but may influence field-level performance.

Future investigations should focus on elucidating the molecular mechanisms underlying the observed changes. High-throughput transcriptomic and proteomic approaches could identify specific genes and signaling pathways influenced by bacterial infection (Walduck et al., 2004). Such data would clarify whether reproductive enhancement arises from hormonal regulation, nutrient provisioning, or immune suppression (Groothuis et al., 2019). Expanding the range of bacterial strains tested will also be critical, as some microbes may confer synergistic benefits while others impose hidden costs.

Field validation represents another priority. Experiments should examine whether symbiotic bacteria-inoculated parasitoids maintain superior performance under natural conditions characterized by fluctuating temperatures, plant chemical defenses, and pathogen exposure (Fontana et al., 2021). Additionally, long-term population dynamics need to be studied to assess whether enhanced female ratios translate into sustainable increases in parasitoid populations (van Nouhuys and Laine, 2008). Finally, integrating microbial manipulation with selective breeding of *A. gifuensis* could create robust lines optimized for large-scale biological control programs.

## 5 Conclusion

This study provides strong evidence that exposure to specific symbiotic bacteria can significantly modulate both the biological performance and physiological traits of *Aphidius gifuensis*, enhancing their effectiveness as biological control agents. Among the strains tested, *Bacillus subtilis* improved parasitism rate and emergence, *Exiguobacterium* enhanced parasitism and increased female offspring production, while *Acinetobacter radioresistens* exerted concentration-dependent effects on emergence and antioxidant enzyme activity. These findings highlight that symbiotic bacterial associations are crucial in shaping parasitoid performance by influencing both reproductive success and enzymatic regulation, which are central to effective biological control strategies. The novelty of this work lies in linking symbiotic bacterial exposure with measurable changes in host reproductive traits and enzyme activities, enriching our understanding of symbiont-parasitoid interactions. However, the controlled laboratory conditions and limited bacterial strains examined suggest that further research is needed. Field validation, exploration of molecular mechanisms, and screening of additional microbial taxa are critical next steps to fully exploit these associations. This study underscores the potential of symbiotic bacteria as natural enhancers of parasitoid efficacy, contributing to more sustainable and environmentally friendly management of *Myzus persicae* in tobacco ecosystems.

## Data Availability

The raw data supporting the conclusions of this article will be made available by the authors, without undue reservation.
